# Efficacy and Safety of Jiedu Tongluo Therapy for Diabetic Kidney Disease Treatment: A Systematic Review and Meta-Analysis

**DOI:** 10.1155/jdr/4180944

**Published:** 2024-11-26

**Authors:** Yuxin Liu, Xiaoling Shang, Hongliang Wu, Ze He

**Affiliations:** ^1^Basic Medical School, Changchun University of Chinese Medicine, Changchun 130117, China; ^2^Vocational Education Teaching and Research Training Center, Jilin Provincial Institute of Education, Changchun 130117, China; ^3^Affiliated Hospital of Changchun University of Traditional Chinese Medicine, Changchun 130117, China

**Keywords:** diabetic kidney disease, Jiedu Tongluo therapy, meta-analysis

## Abstract

**Background:** No comprehensive meta-analysis has evaluated the efficacy and safety of the protective effect of Jiedu Tongluo Therapy on the kidney of DKD until now. This meta-analysis covers this gap in knowledge.

**Methods:** We have conducted an extensive search of databases, including CNKI, Wanfang, PubMed, and Web of Science. The selection was based on conventional treatment, including information and education on DKD, blood glucose, hypertension control methods, and lifestyle. The control group was composed of conventional western medicine or proprietary Chinese medicine, and the experimental group was composed of Jiedu Tongluo therapy controlled trials (RCTs) between 2003 and 2023. R 4.1.0 software was used to perform statistical analysis.

**Results:** A total of 1871 patients from 19 RCTs were analyzed. Meta-analysis results showed that the Jiedu Tongluo therapy was effective in improving clinical efficacy (OR = 2.47, 95% CI [1.94, 3.15], *I*^2^ = 0%), and these trials were more effective in reducing Scr (MD = −19.81, 95% CI [−27.64, −11.97], *p* < 0.01), BUN (MD = −0.70, 95% CI [−1.13, −0.27], *p* < 0.01), UAER (MD = −29.97, 95% CI [−37.33, −22.61], *p* < 0.01), FBG (MD = −0.85, 95% CI [−1.22, −0.47], *p* < 0.01), and certain medication safety (OR = 0.75, 95% CI [0.27, 2.11]).

**Conclusions:** For treating diabetic kidney disease, TCM-based Jiedu Tongluo therapy showed optimal clinical efficacy and safety. However, further rational experiments are needed to validate the above conclusions.

## 1. Introduction

Diabetes-related kidney disease (DKD) is a common chronic consequence of diabetes and a primary cause of uremia. DKD affects 30%–40% of diabetic people [1]. In 2025, the number of patients with DKD will significantly increase [2]. DKD has been observed to affect 21.8% of T2DM patients in China [3]. There is a considerable socioeconomic and public health burden on DKD. Therefore, finding effective therapies for preventing and treating DKD is essential.

Current treatment strategies for DKD focus on controlling blood sugar, blood pressure, and lipid metabolic disorders or use the renin–angiotensin–aldosterone system (RAAS) [4]. It has been clinically proven that these treatments were effective in delaying the progression of DKD. However, it is still difficult to prevent the progression of the disease course, and there is still a lack of targeted and specific treatments. Therefore, finding an effective and safe method to delay the development process of DKD is critical. In China, kidney illness has been treated for countless years using traditional Chinese medicine (TCM). Based on ancient Chinese medicine literature, DKD comes under the categories of “Shen Xiao,” “Xia Xiao,” and “Shen Ke” in Chinese medicine [5]. These names of TCM illustrate the results of excessive consumption or impairment of kidney function. What are the causes of kidney injury? Professor Zheng Nan proposed that “toxic damage to blood vessels of the kidney” is crucial to understanding how DKD develops. He also considers that pathogenic factors acting on the kidneys for a long time will produce toxic substances that stay in the kidneys' meridians and cause damage. Therefore, Jiedu Tongluo should be used as an important foundation and strategy for the treatment of this disease.

Internationally, we usually use the word “toxin” to refer to the Chinese medicine “Du,” but the two are not the same. The scope of “Du” is much larger than that of “toxin,” covering three levels of pathogenic factors, pathological states, and pathological products. It can contain the sum of all substances, information, and energy harmful to the human body. In simple terms, it can be thought of as a harmful substance produced by some pathological processes, such as inflammatory cytokines, endotoxins, lipid peroxidation, reactive oxygen species (ROS), and advanced glycation end products (AGE). It can also be regarded as a pathological state, such as overexpression of the MMP family and abnormal increase or excessive deposition of extracellular matrix (ECM) caused by various lesions and damage, and eventually lead to functional changes of cells, tissues, and organs. Research proved that toxicity is always the major cause of microvascular injury. “Luo” disease in TCM is very similar to microvascular disease, which expresses the pathological state of microcirculation dysfunction and poor blood flow in tissues and organs. For example, in the progression of DKD, the renal collaterals can be seen as small blood vessels, microvessels, including microcirculation, especially the glomerular capillary loops, distributed in the renal region. Under physiological conditions, the kidney collaterals have the function of assisting the kidney to eliminate excess water and metabolic waste in the blood. In pathological conditions, once the kidney collateral stasis, it can represent various glomerular lesions, such as vascular necrosis, mesangial matrix increase, renal balloon wall destruction, lumen stenosis, or even obstruction. As the strongest known vascular permeability factor, VEGF affects the glomerular filtration barrier. Aggravating the extravasation of blood components leads to the accumulation of glomerular extracellular matrix, the thickening of the basement membrane, the progression of glomerular sclerosis and renal interstitial fibrosis, and then the emergence of massive proteinuria. This is highly consistent with the pathological state of “blood does not follow the meridian, overflows outside the pulse, and obstructs the kidney collaterals” in TCM. Based on the characteristics of glomerular lesions, TCM believes that the efficacy of grass and wood products for promoting blood circulation and removing blood stasis is difficult to reach the lesion site, and insects should be used to enter the collaterals and clear collaterals. Therefore, in the treatment of TCM, pangolin, turtle beetle, scorpion, leeches, and other conventional drugs such as *Angelica sinensis*, chuanxiong, *Salvia miltiorrhiza*, peach kernels, and safflower are often used in coordination to achieve the role of promoting blood circulation and clearing collaterals. The Yun Ma research group found [6] that the Huayu Tongluo formula composed of chuanxiong, Salvia salvia, scorpion, leechi, and dilong can effectively inhibit the overexpression of VEGF in rat renal tissue and make up for the deficiency of VEGF-targeted inhibitors that may cause kidney injury [7]. In addition, under pathological conditions, various reasons cause the synthesis and secretion of ECM to increase, and then excessive deposition in the glomeruli, which is the main link leading to glomerular sclerosis, similar to the formation process of “Du” in Chinese medicine. Therefore, some Chinese medicines with detoxification effects such as *Tripterygium wilfordii*, Shanglu, morning glory, rhubarb, and hazel flower will be used to improve the state of the “Du” knot. It was found that *Tripterygium wilfordii* could reduce urinary protein and TGF-*β*1 expression in the blood of adriamycin nephropathy model rats, thereby reducing ECM aggregation, delaying glomerulosclerosis, and protecting renal function [8]. By intervening in the expression of key signal molecules Smad3 and p-Smad2/3 in the TGF-*β*1/Smad signaling pathway, *Tripterygium* glycosides can reduce the deposition of ECM component Col-1 in renal tissue and reduce the expression of *α*-SMA, thereby protecting renal function [9]. Shanglu water decoction can inhibit the expression of FN and LN of ECM components, upregulate the expression of MMP-2, and delay the progression of glomerulosclerosis [10]. The combination of Yizhiren–Wuxin can effectively reduce the mRNA expressions of laminin, fibronectin, and Collagens I and IV of ECM-related fibrosis genes, thereby improving the kidney function of DKD mice [11].

In summary, Professor Nan Zheng combined the above drug characteristics, according to the pathological evolution characteristics of DKD, and summed up the treatment of “JieDuTongLuo.” Using it to improve glomerular function and prevent and cure DKD, on the one hand, is an innovation based on the theory of TCM and the theory of collaterals and develops the advantages and characteristics of national medicine. On the one hand, providing ideas and methods for clinical prevention and treatment of DKD in the future has far-reaching benefits. Many studies have been published over the last several years, which provided strong evidence for the pathogenic mechanisms and the effectiveness of the corresponding treatment method. However, only a small number of research have thoroughly evaluated the effectiveness and safety of the therapy. In order to assess the effectiveness and safety of this approach and to offer evidence-based medical references, we did this systematic review and meta-analysis.

## 2. Methods

### 2.1. Search Strategy

We performed comprehensive searches on databases, such as Wanfang, CNKI, VIP, Web of Science, Cochrane Library, and PubMed, from June 2003 to April 2023. The search keywords include diabetic kidney disease, Xiao Ke nephropathy, Jiedu Tongluo, and Chinese herbal medicine. The searches were performed using subject terms and were limited to studies conducted in humans, with no restrictions on languages. The compilation of manual searches of conference reports was used as supporting data.

### 2.2. Study Selection

Studies might be included if they matched all of the following requirements: (i) patients who were consistent with a diagnosis of diabetic nephropathy and were staged using the Mogensen method, regardless of the stage of DKD [12]; (ii) relevant RCT experiments; (iii) the intervention of DKD included based on the conventional treatment (diabetes and nephropathy health education, lifestyle intervention, blood glucose control, and blood pressure lowering), either standard western or Chinese medication was used to treat the control group, and the experimental group only used TCM with Jiedu Tongluo or combined western medicine treatment; and (iv) the results contained one or more of the following: total clinical effectiveness, safety, adverse effects, serum creatinine, blood urea nitrogen, fasting blood glucose, and urine albumin excretion rate, and at least one item includes serum creatinine, blood urea nitrogen, or urine albumin excretion rate.

### 2.3. Exclusion Criteria

Excluded criteria are as follows: non-RCTs; duplicate publications; and literature containing incomplete information, errors, or inaccessible critical information. The patients included suffered other diseases affecting kidney function, and the interventions included other TCM therapies.

### 2.4. Data Extraction

Information about the study's subject characteristics, design, blindness, and randomization was independently gathered by two researchers (e.g., sample size, course of the disease, intervention, medication form, course of treatment, and safety evaluation), and when there were differences of opinion, disagreements were settled following discussion with other investigators.

### 2.5. Data Analysis

The Cochrane Collaboration conducted a meta-analysis using R Version 4.1.0. Tests of heterogeneity were used to evaluate variability in intervention effects. Heterogeneity is considered significant when *I*^2^ is over 40% or *p* < 0.05. If there was considerable heterogeneity, the meta-analysis employed the random effect model. On the contrary, we believe that the combined data showed good homogeneity. The test level was set to 0.05, and continuous data were presented as average difference (MD) with a 95% confidence interval (CI).

### 2.6. Bias Evaluation

We can use the bias risk assessment standards to evaluate the risks and quality of studies [13]. The methods used to create randomly generated sequences were evaluated, including whether the personnel is strictly implemented, whether the blinding has been used, whether the outcome measurement has been completed, whether the positive results are selectively reported, and whether another bias is possible. According to the evaluation criteria, low, unknown, and high risk were judged.

### 2.7. GRADE Level Evaluation

By studying limitations, inconsistency, indirectness, imprecision, and publication bias, the evidence quality of outcome indicators was rated. The RCT evidence quality rating was preset as “high,” “moderate,” “low,” and “very low,” and the RCT evidence quality rating was preset as “moderate,” “low,” and “very low” [14]. The evaluation was completed independently by two researchers, then cross-checked, and in case of disagreement, a third researcher arbitrated.

## 3. Results

### 3.1. Literature Search Results

**Figure 1:**
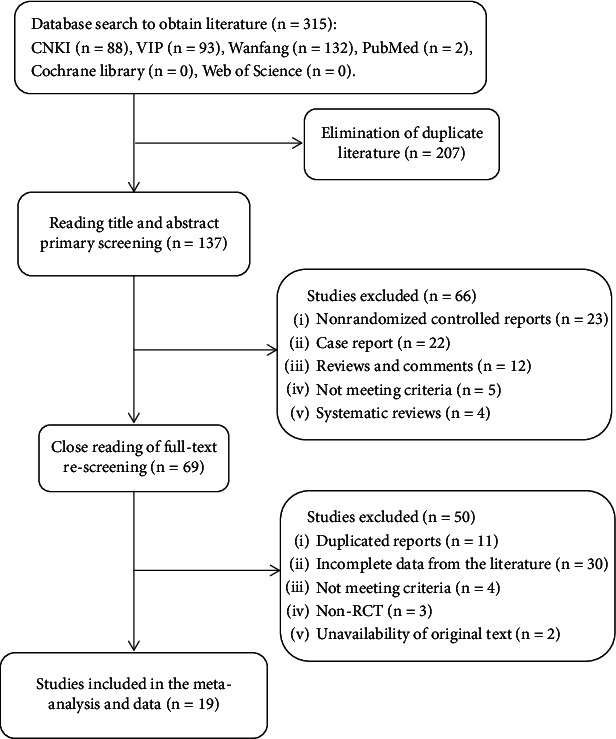
Literature screening process.

By searching for terms and related topics, we completed the inclusion of 315 relevant articles. Among them, there were 313 Chinese literatures and two foreign literatures, which were then strictly screened. First, 207 duplicate literatures were excluded. Second, after reading the title and abstract, 66 literatures that did not meet the research criteria were excluded. Third, after carefully reading the full text, 50 articles were excluded. Finally, 19 articles were included in the study, involving 1871 patients [15–33] (Figure 1 and Table 1). Sample sizes of either the experimental or control groups were 30 or more. The mean age range in the experimental group and control group was 42–59.24 and 42.5–59.3 years, respectively. The age range was generally matched between the two groups. Study courses range from 4 to 12 weeks, and two of these studies did not describe course length.

### 3.2. Bias Evaluation Results

**Figure 2:**
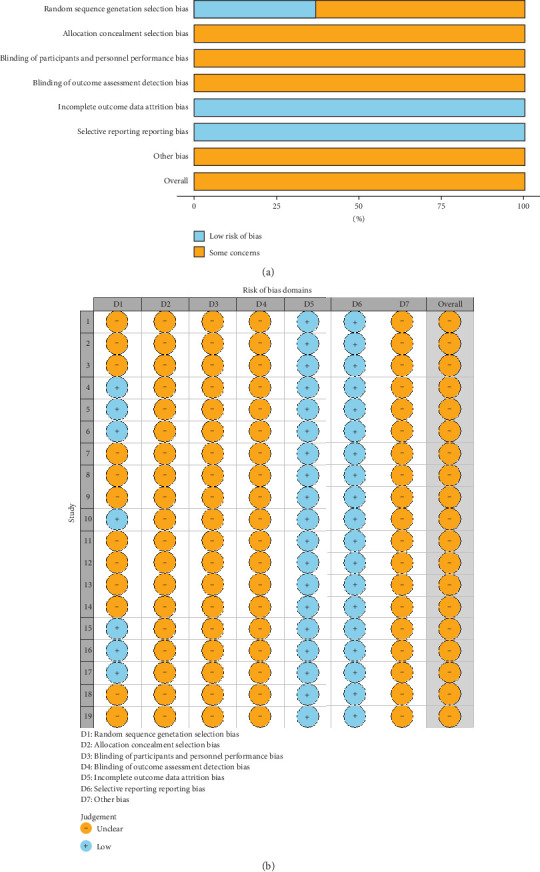
Distribution of the included RCTs' bias risk. (a) Risk of bias chart and (b) sum of risk of deviation. D1 = random sequence generation (selection bias), D2 = allocation concealment (selection bias), D3 = participant and personnel blindness (implementation bias), D4 = result evaluation.

**Figure 3:**
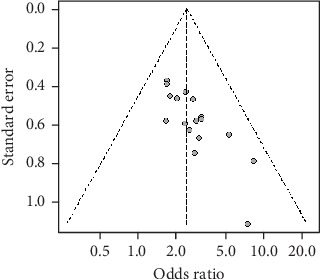
Funnel diagram for the efficacy of Jiedu Tongluo therapy for DKD.

Briefly, seven studies [18–20, 24, 29–31] mentioned the use of a particular randomization method and were therefore evaluated as low risk. Twelve articles [15–17, 21–23, 25–28, 32–33] only mentioned randomized grouping and did not mention the particular method and were considered as unclear risk. All of the trials were regarded as low risk and had distinct outcome measures. The risk was minimal since there were no duplicate publications or disclosed biases in the research, and the remaining biases were unidentified and rated as ambiguous. All information was provided in full and was compared across groups (Figure 2). Software R Version 4.1.0 draws a funnel plot for publication bias detection, which shows basic symmetry and has a certain significant bias (Figure 3).

### 3.3. GRADE Level Evaluation Results

A total of five outcome measures were included in the meta-analysis of 19 articles, among which one outcome measure was rated as low-level GRADE, and the remaining four outcome measures were rated as extremely low-level GRADE (Table 2).

### 3.4. Meta-Analysis Results

#### 3.4.1. Effect on Clinical Efficiency

**Figure 4:**
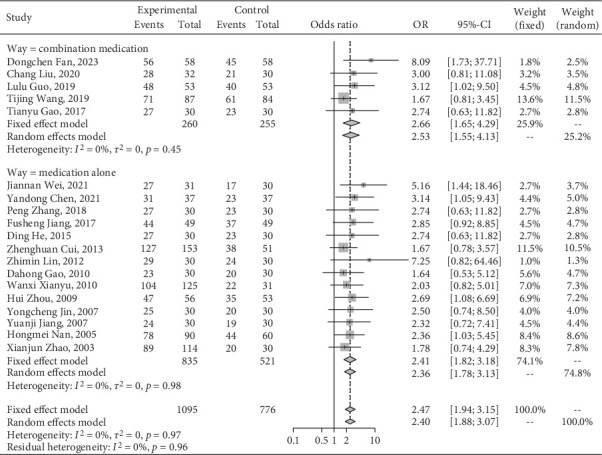
The efficacy of Jiedu Tongluo therapy for DKD according to the clinical interventions.

Nineteen studies [15–33] mentioned the clinical total response rate of the Jiedu Tongluo therapy. TCM western medication oral agents were given in five studies [15, 18–20, 22]. There are 1095 and 776 patients in the experimental and comparison groups, respectively. No interstudy heterogeneity was observed (*I*^2^ = 0%, *p* = 0.97), and the clinical effective rate was higher in the experimental group (OR = 2.47, 95% CI [1.94, 3.15], *I*^2^ = 0%). After subgroup analysis, the effective rate of TCM oral agents combined with western medicine treatment was lower than that of oral treatment with TCM (Figure 4).

#### 3.4.2. Effect on FBG

**Figure 5:**
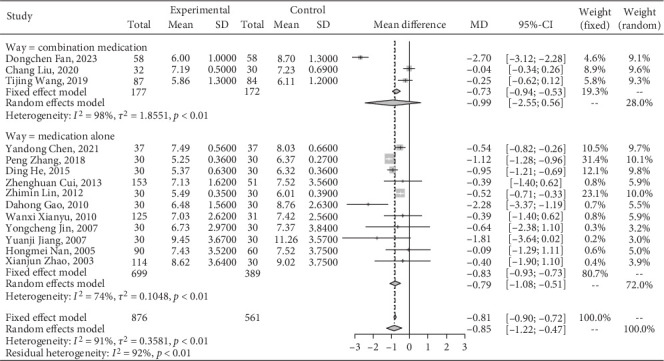
A meta-analysis of Jiedu Tongluo therapy for FBG according to the clinical interventions.

Fourteen studies [15, 17–18, 20–21, 24–28, 30–33] mentioned FBG. TCM western medication oral agents were given in three studies [15, 18, 20]. There were 876 and 561 patients in the experimental and comparison groups, respectively. There was a great heterogeneity among the articles (*I*^2^ = 91%, *p* < 0.01), and Jiedu Tongluo therapy can effectively reduce FBG (MD = −0.85, 95% CI [−1.22, −0.47], *p* < 0.01). They can effectively reduce FBG in both simple TCM oral agent treatment (MD = −0.79, 95% CI [−1.08, −0.51], *p* < 0.01) and TCM oral agent combined with western medicine treatment (MD = −0.99, 95% CI [−2.55, 0.56], *p* < 0.01) (the difference was not statistically significant) after subgroup analysis. Decreased heterogeneity of oral Chinese medicine agents treatment is observed (*I*^2^ = 74%, *p* < 0.01). The effect of oral Chinese medicine is greater than that of oral Chinese medicine agents combined with western medicine (Figure 5).

#### 3.4.3. Effect on Scr

**Figure 6:**
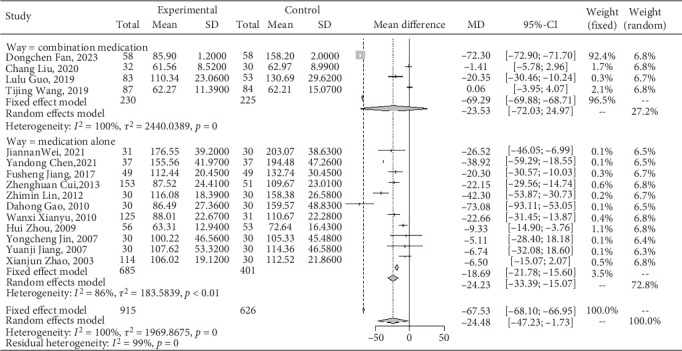
A meta-analysis of Jiedu Tongluo therapy for Scr according to the clinical interventions.

A total of 15 studies [15–20, 23, 25–31, 33] mentioned Scr. TCM and western medicine oral agents combination in 3 of the 14 studies [18–20]. There are 857 and 568 patients in the experimental and comparison groups, respectively. The overall study has shown that there was a great heterogeneity among the articles (*I*^2^ = 91%, *p* < 0.01), and Jiedu Tongluo Therapy can effectively reduce Scr (MD = −19.81, 95% CI [−27.64, −11.97], *p* < 0.01). After subgroup analysis, they can effectively reduce Scr both of simple TCM oral agent treatment (MD = −24.23, 95% CI [−33.39, −15.07], *p* < 0.01) and TCM oral agent combined with western medicine treatment (MD = −5.69, 95% CI [−13.97, 2.59], *p* < 0.01) (the difference was not statistically significant). The heterogeneity of oral Chinese medicine agent treatment is decreased (*I*^2^ = 86%, *p* < 0.01). The effect of oral Chinese medicine agents combined with western medicine is greater than that of oral Chinese medicine (Figure 6).

#### 3.4.4. Effect on BUN

**Figure 7:**
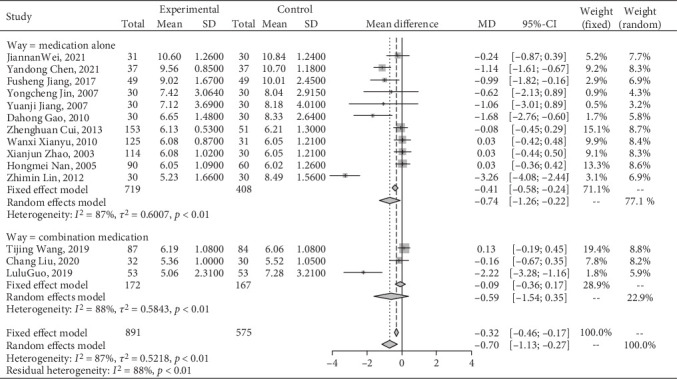
A meta-analysis of Jiedu Tongluo therapy for BUN according to the clinical interventions.

Fourteen studies [16–20, 23, 25–28, 30–33] mentioned BUN. TCM western medication oral agents were given in three studies [18–20]. There are 891 and 575 patients in the experimental and comparison groups, respectively. The overall study shows that there is great heterogeneity among the articles (*I*^2^ = 88%, *p* < 0.01), and Jiedu Tongluo Therapy can effectively reduce BUN (MD = −0.70, 95% CI [−1.13, −0.27], *p* < 0.01). After subgroup analysis, they can effectively reduce BUN in both simple TCM oral agent treatment (MD = −0.74, 95% CI [−1.26, −0.22], p < 0.01) and TCM oral agent combined with western medicine treatment (MD = −0.59, 95% CI [−1.54, 0.36], *p* < 0.01). The heterogeneity of oral Chinese medicine agents combined with western medicine treatment is decreased (*I*^2^ = 86%, *p* < 0.01). The effect of oral Chinese medicine is greater than that of oral Chinese medicine agents combined with western medicine (Figure 7).

#### 3.4.5. Effect on UAER

**Figure 8:**
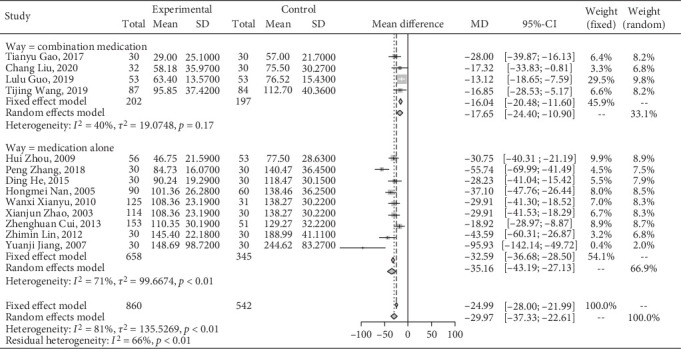
A meta-analysis of Jiedu Tongluo therapy for UAER according to the clinical interventions.

A total of 13 studies [18–22, 24–26, 28–29, 31–33] mentioned UAER. TCM western medication oral agents were given in four studies [18–20, 22]. There are 860 and 542 patients in the experimental and comparison groups, respectively. The overall study has shown that there was a great heterogeneity among the articles (*I*^2^ = 81%, *p* < 0.01), and Jiedu Tongluo therapy can effectively reduce UAER (MD = −29.97, 95% CI [−37.33, −22.61], *p* < 0.01). After subgroup analysis, they can effectively reduce UAER both of simple TCM oral agent treatment (MD = −17.65, 95% CI [−24.40, −10.90], *p* < 0.01) and TCM oral agent combined with western medicine treatment (MD = −35.16, 95% CI [−43.19, −27.13], *p* < 0.01). The heterogeneity of oral Chinese medicine treatment is decreased (*I*^2^ = 40%, *p* = 0.17). The effect of oral Chinese medicine is greater than that of oral Chinese medicine agents combined with western medicine (Figure 8).

### 3.5. Adverse Reactions

**Figure 9:**
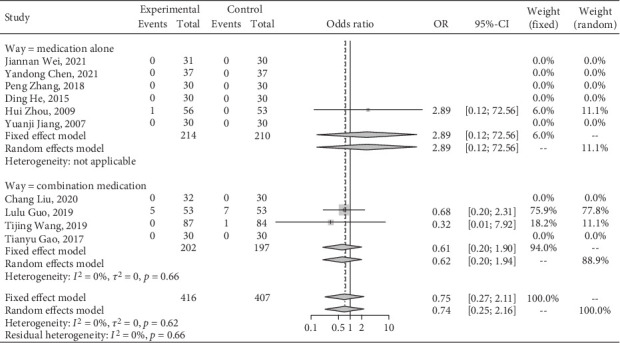
A meta-analysis of the adverse reactions to Jiedu Tongluo therapy according to the clinical interventions.

Seven studies [16–18, 21–22, 24, 31] in the included literature had no adverse effects, nine studies [15, 23, 25–28, 30, 32–33] were not reported, and three studies [19–20, 29] mentioned adverse reactions. In total, 14 patients reported adverse events. Six patients in the treatment group experienced adverse events. Among them, two cases experienced lacking strength, one case of headache, two cases of gastrointestinal reaction loss of appetite, and one case of laxativeness. Meanwhile, eight patients in the control group had side effects. Among them, two cases lacked strength, two cases with headaches, three cases of gastrointestinal reaction loss of appetite, and one case of excitant dry cough. The heterogeneity test revealed no interstudy heterogeneity (*I*^2^ = 0%, *p* = 0.62), and no difference was identified in safety between Jiedu Tongluo therapy and conventional chemical drug therapy (OR = 0.75, 95% CI [0.27, 2.11]) (Figure 9).

## 4. Discussion

As a serious consequence of diabetes mellitus and end-stage renal disease, DKD has been recognized. However, to date, only blocking the RAAS and multidisciplinary treatments are effective treatments. It has recently been reported that SGLT2 may inhibit DKD progression [34], and in 2019, their inhibitors were included as the most effective new medication for the therapy of DKD [35]. However, they still could not prevent patients from developing ESKD successively. As a result, more effective prevention measures are urgently needed to delay the onset of DKD. TCM has been treating kidney disease for thousands of years, and adverse reactions have been reported less frequently, which may be a more advantageous intervention. In China, many patients use TCM as a complementary and necessary combination therapy to prevent and treat DKD nephropathy. It has been demonstrated that TCM has good clinical benefits in treating DKD. In a meta-analysis, Chinese herbals were more effective in reducing UAER and proteinuria than placebo or ACEI/ARB. Chinese herbal remedies were more successful than a placebo or ACEI/ARB at lowering UAER and proteinuria [36]. Applying Chinese remedies to treat and control DKD is a wise course of action. TCM treats diseases by using the method of treatment to guide the compatibility of medicines, so an effective treatment is very important.

Following decades of clinical investigation and observation, Professor Zheng Nan has discovered that DKD is a chronic disease of yin and yang deficiency resulting in different pathological products such as phlegm, dampness, and blood stasis. They are entangled and developed into the internal accumulation of poisonous pathogenic factors to influence the circulation of qi-blood, and the movement of body fluid will lead to damage to the renal collaterals, the kidneys are injured, and kidney qi is exhausted, which may be the main path mechanism of DKD. That is why it is said that kidney collaterals are impaired by toxins. Therefore, Jiedu Tongluo is regarded as the source and crucial concept for curing this illness [37].

Jiedu Tongluo treatment for DKD greatly decreased the signs of renal damage, according to the study's findings, such as Scr, BUN, and UAER, reduced FBG, and improved overall clinical efficiency. Jiedu Tongluo Chinese medicine oral agents are superior to Jiedu Tongluo therapy adjuvant to western medicine in controlling the aspects of overall clinical efficiency, FBG, BUN, and UAER. But Jiedu Tongluo therapy adjuvant to western medicine has a superiority more than Jiedu Tongluo Chinese medicine oral agent in Scr. Regarding the safety of medicines, Jiedu Tongluo therapy did not have any significant adverse effects. Although there is different compatibility of Chinese herbs in all the studies in the included literature, Jiedu Tongluo therapy is the main treatment method. The studies that evaluated FBG, Scr, BUN, and UAER showed significant heterogeneity. It indicated that blood glucose and kidney function are also influenced by emotional fluctuation, the stress of discrepancy, the rhythm of life, exercise intensity, and diet. In addition, the high heterogeneity of studies came from that disease course stage, syndrome classification of TCM, compatibility, and dosages of Chinese herbs, age, disease duration, and tolerance, which may be the important cause of this high heterogeneity. Therefore, in order to conduct a more thorough and reproducible study on the usage of Jiedu Tongluo therapy, an effective lifestyle intervention is urgently needed.

This meta-analysis provides a detailed evaluation of the effectiveness of Jiedu Tongluo therapy in the treatment of DKD, which is different from previous studies evaluating the treatment of DKD with a single Chinese medicine or a single prescription. The data sources of this study will be more abundant, and the data of multiple independent studies on the treatment of DKD with different Chinese medicines and formulas mainly based on this method will be integrated. This study breaks the limitations of previous meta-analyses and provides more general and representative conclusions for TCM treatment of DKD. Although the specific mechanism of Jiedu Tongluo therapy is not yet clear, the evaluation of its effectiveness and safety in this meta-analysis provides evidence support for the formulation of TCM prevention and treatment strategies for DKD. At the same time, it also provides clues and directions for in-depth exploration of the biological mechanism of Jiedu Tongluo therapy and collateralization in the treatment of diabetic kidney disease and promotes the progress of basic research.

This research approach is consistent with the “holistic concept” and “syndrome differentiation and treatment” principles followed by TCM therapy, which emphasizes individualized diagnosis and treatment characteristics of patients. It is worth noting that in the process of data collection, according to the TCM diagnosis and treatment mode, we found that the TCM syndrome differentiation of qi and yin deficiency, liver kidney yin deficiency, and spleen kidney yang deficiency exists in most DKD patients of different stages, accompanied by pathological states of varying degrees of “toxic knot” and “Luo” diseases. These patients may benefit the most from Jiedu Tongluo therapy. In future clinical research, we should focus on the pathological characteristics of the three TCM syndrome types mentioned above; clarify the evolution rules of different subtypes in the development of DKD; further optimize the clinical application of detoxification and meridian therapy; explore the combination, dosage, and mechanism of action of different detoxification and meridian drugs; develop personalized treatment plans for different syndrome types; and provide more effective TCM strategies for the treatment of DKD.

In addition, judging from the main conclusions of the meta-analysis included in this study, the clinical efficacy of detoxification and collateralization therapy in the treatment of DKD is worthy of affirmation. However, GRADE was used to evaluate the evidence quality of a single outcome indicator, and it was found that the evidence quality ratings of the outcome indicators involved in the relevant meta-analysis were both low and very low, suggesting that the real effect was likely to be different from the expected effect. The main reason is the limitation of the research, which is embodied in randomization, blind method, assignment hiding, and selective reporting. The secondary reasons were publication bias, inconsistencies, and inaccuracies, including incomplete funnel plot symmetry or publication bias not investigated due to fewer included studies, large clinical and statistical heterogeneity of the original study, small sample size, and wide 95% CI range. This greatly reduces the reliability of the conclusions of relevant meta-analyses. The results of the study showed that the treatment of DKD has certain advantages, especially in the improvement of clinical total effective rate and outcome indicators such as FBG, Scr, BUN, and UAER. However, due to the uneven quality of relevant meta-analysis methodology and low evidence strength, the results of this study should be treated with caution. For future meta-analyses, we suggest that the scheme design should be standardized before the study. In particular, attention should be paid to the preparation of meta-analysis plans in advance, the preregistration of research schemes, a comprehensive literature search strategy, the provision of a list of excluded literatures and reasons for exclusion, and the risk of bias and its impact on the results of meta-analysis. And then make a scientific and comprehensive meta-analysis. In view of the high heterogeneity, we suggest that in the meta-analysis of DKD treated with Jiedu Tongluo therapy in the future, subgroup analysis should be conducted strictly according to intervention population, intervention measures, treatment cycle, and follow-up time to reduce bias. In the evaluation of outcome indicators, subjective indicators (such as TCM clinical symptoms and syndrome scores) and long-term efficacy (such as patient survival rate) can be added, which should be fully considered in the future.

### 4.1. Limitations

Our study showed that despite Jiedu Tongluo therapy to control DKD progress effectively. There are still some gaps in the study that should be explored for additional improvements. Firstly, the quality of the RCT was low. The blinding and allocation concealment were not mentioned in any of the literature included in this study. This was a limitation of many clinical trials. Second, the heterogeneity is high among some outcome indicators. The heterogeneity was not significantly altered after subgroup analysis because the baseline values and parameters varied widely between the studies and the type of intervention. Third, there may be a potential source of bias for data extracted as an unblinded method. Fourth, it was inevitable for some linguistic biases, with only Chinese and English literature inclusion. Lastly, most of the studies were not reported by gender, so we were unable to determine whether gender affected the outcome. In addition, our results have shown that Chinese patients were the main subjects of the experimental study, and it has some influence on the promotion and use of Jiedu Tongluo therapy in other countries, which has given rise to a lack of literature.

This study indicates that Jiedu Tongluo therapy had an effect and is possibly better than western medicine treatment alone. Adhere to treatment according to the syndrome differentiation of TCM, which effectively targeted different stages and types of disease to match Chinese herbs. It could provide evidence orientation and treatment strategies for TCM to exert its pharmacological effects in the treatment of DKD. In this study, the pharmacological effect of relevant TCM has not been deeply analyzed, and the included subjects were mainly Chinese. Future large samples, multicentre, and rigorous clinical trials were required to further verify our findings.

## Figures and Tables

**Table 1:** Baseline features of the included patients.

**Author(s), year**	**Sample size (T/C)**	**Age (years)**		**Course of disease (years)**		**Sex ratio (men/women)**		**Interventions**		**Treatment duration**
		**E**	**C**	**E**	**C**	**E**	**C**	**E**	**C**	
Fan, 2023 [15]	58/58	42.0 ± 1.1	42.5 ± 1.0	11.0 ± 1.2	10.5 ± 1.0	31/27	30/28	N+Jiedu Tongluo decoction+irbesartan tablets	N+irbesartan tablets	90 days
Wei, 2021 [16]	31/30	—	—	—	—	20/11	19/11	N+Jiedu Tongluo decoction	N	12 weeks
Chen, 2021 [17]	37/37	—	—	—	—	21/16	25/12	N+Jiedu Tongluo decoction	N	3 months
Liu, 2020 [18]	32/30	54.50 ± 9.90	53.80 ± 9.70	—	—	20/12	16/14	N+Jiedu Tongluo decoction+irbesartan tablets	N+irbesartan tablets	12 weeks
Guo, 2019 [19]	53/53	57.98 ± 4.33	58.22 ± 4.26	5.21 ± 2.02	5.02 ± 1.98	25/28	23/30	N+Jiedu Tongluo decoction+valsartan tablets	Valsartan dispersible tablets	2 months
Wang, 2019 [20]	87/84	59.24 ± 10.35	61.73 ± 11.05	6.12 ± 5.24	5.93 ± 4.98	42/45	41/43	N+Jiedu Tongluo decoction+irbesartan tablets	N+irbesartan tablets	2 months
Zhang, 2018 [21]	30/30	—	—	—	—	20/10	23/7	N+Jiedu Tongluo decoction	D+Liuwei Dihuang decoction	12 weeks
Gao and Tian, 2017 [22]	30/30	52.00 ± 8.10	53.00 ± 7.60	3.10 ± 2.20	3.20 ± 2.00	18/12	16/14	N+Jiedu Tongluo capsule+losartan potassium tablets	N+losartan potassium tablets	12 weeks
Jiang and Nan, 2017 [23]	49/49	60.10 ± 3.60	60.30 ± 3.40	4.50 ± 1.40	4.30 ± 1.20	21/28	20/29	N+Jiedu Tongluo decoction	N+calcium dobesilate capsules	60 days
He, 2015 [24]	30/30	52.4 ± 10.84	48.84 ± 10.81	7.84 ± 4.64	9.25 ± 5.49	25/5	26/4	N+Jiedu Tongluo decoction	N+ShenQiDiHuang decoction	12 weeks
Cui, 2013 [25]	153/51	55.10 ± 7.20	54.30 ± 6.80	9.91 ± 1.30	8.50 ± 5.10	—	—	N+Jiedu Tongluo decoction	N+benazepril tablets	3 months
Lin, 2012 [26]	30/30	—	—	—	—	15/15	12/18	N+Xuantu of Dan	N+enalapril maleate tablets	—
Gao, 2010 [27]	30/30	54.70 ± 11.78	57.30 ± 12.14	8.85 ± 2.62	8.92 ± 2.53	17/13	16/14	N+Jiedu Tongluo capsule	N	30 days
Xianyu, 2010 [28]	125/31	57.60 ± 5.80	58.50 ± 6.40	9.20 ± 3.80	8.60 ± 3.30			N+Jiedu Tongluo decoction	N+benazepril tablets	3 months
Zhou, 2009 [29]	56/53	55.86 ± 8.15	53.61 ± 10.63	8.36 ± 4.53	8.42 ± 3.91	22/34	20/33	N+Jiedu Tongluo decoction	N+losartan potassium tablets	12 weeks
Jin and Nan, 2007 [30]	30/30	—	—	—	—	10/20	13/17	N+Jiedu Tongluo capsule	N+gliquidone tablets	8 weeks
Jiang, 2007 [31]	30/30	52.61 ± 8.79	53.37 ± 8.18	7.98 ± 3.83	7.73 ± 3.65	15/15	14/16	N+Jiedu Tongluo granules	N+gliquidone tablets	8 weeks
Nan, Piao, and He, 2005 [32]	90/60	56.20 ± 5.40	55.30 ± 3.20	9.30 ± 2.40	8.80 ± 2.30	51/39	33/27	N+Jiedu Tongluo capsule	N	90 days
Zhao and Deng, 2003 [33]	114/30	58.20 ± 6.40	56.50 ± 7.20	8.30 ± 3.20	7.80 ± 3.30	7.8 ± 3.3	16/14	N+Jiedu Tongluo granules	N+benazepril hydrochloride tablets	—

*Note:* N, conventional western medicine for DKD, including blood glucose control, blood pressure lowering, lipid regulation, and other conventional treatments; E, experimental group; C, control group; —, not mentioned.

**Table 2:** GRADE classifications.

**Outcome indicators**	**The number of RCTs**	**Limitations**	**Inconsistency**	**Indirectness**	**Imprecision**	**Publication bias**	**Experimental group**	**Control group**	**Effect quantity**	**The evidence level**
Efficacy	19	Serious	No serious inconsistency	No serious indirectness	No serious imprecision	Likely	1095	776	OR = 2.47, 95% CI (1.94, 3.15)	Low
FBG	14	Serious	Very serious inconsistency	No serious indirectness	No serious imprecision	Likely	876	561	MD = −0.85, 95% CI (−1.22, −0.47)	Very low
Scr	14	Serious	Very serious inconsistency	No serious indirectness	No serious imprecision	Likely	915	626	MD = −19.81, 95% CI (−27.64, −11.97)	Very low
BUN	14	Serious	Very serious inconsistency	No serious indirectness	No serious imprecision	Likely	891	575	MD = −0.70, 95% CI (−1.13, −0.27)	Very low
UAER	13	Serious	Very serious inconsistency	No serious indirectness	No serious imprecision	Likely	860	542	MD = −29.97, 95% CI (−37.33, −22.61)	Very low

*Note: I* ^2^ > 50%, which represents seriousness. *I*^2^ > 75%, which represents extreme seriousness.

## Data Availability

The previously published research and datasets that the RCT data for this meta-analysis are based on have been cited. Upon request, the corresponding author will provide the processed data.
