# Supplementary material for: Efficacy and Safety of Jiedu Tongluo Therapy for Diabetic Kidney Disease Treatment: A Systematic Review and Meta-Analysis
Source: J Diabetes Res. 2024 Nov 26;2024:4180944. doi: 10.1155/jdr/4180944 (PMC11614504; doi:10.1155/jdr/4180944)
Supplement: Supporting Information — Additional supporting information can be found online in the Supporting Information section. PRISMA 2020 checklist [38]. Data source and search strategy details [39]. [file 4180944.f1.zip › Supplementary Document.docx]

**Supplementary Document：** Search terms used in literature search.

Wanfang(132):

Subject:(*clinical*) and Keyword:(*diabetic nephropathy or Diabetic kidney disease or Xiaoke nephropathy*) and Keyword:(*jiedu* and *tongluo*)

CNKI(88):

SU%='*diabetic nephropathy'* *'*jiedu*'*'*tongluo*' OR SU%='*Diabetic kidney disease*' *'*jiedu*'*'*tongluo*' ORSU%='*Xiaoke nephropathy*' *'*jiedu*'*'*tongluo*'

VIP(93):

T=*clinical* AND K=*diabetic nephropathy* AND *jiedu* AND *tongluo* OR T=*clinical* AND K=*diabetic kidney disease* AND *jiedu* AND *tongluo* OR T=*clinical* AND K=*Xiaoke nephropathy* AND *jiedu* AND *tongluo*

Pubmed(2):

Search: (*jiedutongluo*) AND *diabetic Kidney Disease*

Web of Science(0):

Search: *jiedutongluo*

Cochrane Library(0):

Basic Search Title Abstract Keyword:*jiedutongluo*
